# The Methylation Test Is Highly Sensitive for HPV‐Associated Endocervical Adenocarcinoma and Could Be Helpful as a Cytological Ancillary Test in Women With a PAP‐Smear Diagnosis of Severe Glandular Lesion (AGC–NEO+)

**DOI:** 10.1111/cyt.70001

**Published:** 2025-06-13

**Authors:** Iva Kinkorová Luňáčková, Ondrej Ondič, Jana Němcová, Kateřina Černá, Jan Chytra, Jiří Bouda

**Affiliations:** ^1^ Bioptická laboratoř, s.r.o Pilsen Czech Republic; ^2^ Charles University and Charles University Hospital Pilsen, Department of Pathology, Medical Faculty Pilsen Czech Republic; ^3^ Charles University and Charles University Hospital Pilsen, Department of Gynecology and Obstetrics, Medical Faculty Pilsen Czech Republic

**Keywords:** adenocarcinoma, cervical cancer screening, cervix, HPV, methylation test, papanicolaou test, pregnancy

## Abstract

**Objective:**

The limitations of PAP smears in diagnosing severe cervical glandular lesions motivate the development of ancillary methods to facilitate their detection. This prospective cytology–histology and molecular study aims to investigate methylation test performance by establishing the test sensitivity and its relevance to patient management.

**Method:**

LBC samples were prospectively acquired after 3 months following the primary conventional PAP‐smear screening diagnosis of AGC–NEO (atypical glandular cells, favour neoplastic) and AIS. An HPV test and methylation test were performed. Corresponding subsequent biopsy reports were collected.

**Results:**

Seven hundred and seventy Pap tests were signed out as AGC–NEO+. Complete study data were available for 85 cases (AIS in 46 cases, EA in 39 cases) that were further analysed. The methylation test was positive in 95.3% (81/85) cases, negative in 3.5% (3/85, AIS in two cases, EA in one case) and unanalysable in one sample (1.2%). HPV genotyping revealed a multi‐infection rate of 29%. The presence of HPV types 16 and 18 was detected in 84% (72/85) of lesions, and HPV type 45 in 12% (10/85).

**Conclusions:**

The methylation test shows a high sensitivity of 95.3% and reliably identifies histologically confirmed AIS+ lesions. This argues for further investigation into its performance characteristics and consideration of its use, especially as a pre‐surgical triaging test in sensitive cases if the AIS+ lesion is suspected in pregnant women or nullipara.

## Graphical abstract

Based on an analysis of 85 endocervical adenocarcinoma (EA) cases with cytology–histology correlation, the authors document a methylation test sensitivity of 95.3% for diagnosing EA. If used as an ancillary test, the methylation test may improve the PAP test's reliability. The authors argue that this may be of special importance to young women.

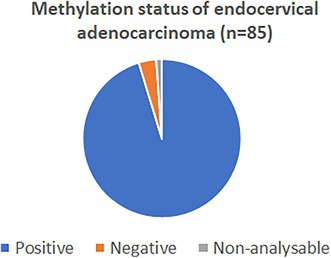

## 1 Introduction

Although the Papanicolaou (Pap) test was initially designed to detect squamous cell carcinoma [1], it can also identify HPV‐associated adenocarcinoma in situ (AIS) and HPV‐associated endocervical adenocarcinoma (EA) with variable sensitivity and specificity [2, 3, 4, 5, 6]. In this regard, the effectiveness of HPV testing has already been investigated [6, 7, 8, 9, 10]. Other methods are under investigation, including selected gene methylation status testing. It is based on detecting the epigenetic mechanism of gene silencing by hypermethylation of the tumour‐suppressor gene promoter region [11, 12].

The aim of this prospective cytology–histology and molecular study is to investigate methylation test performance by establishing sensitivity and its relevance to cervical cancer screening in regard to severe glandular lesions reported cytologically at least as atypical glandular cell‐favour neoplastic (AGC–NEO) with subsequent histological confirmation.

## 2 Materials and Methods

In the setting of a large cervical cancer screening laboratory performing over 800.000 Pap smears per year, a prospective study was carried out between 2016 and 2024 and systematically identified Pap smears harbouring abnormal glandular lesions classified as AGC–NEO, AIS, EA and endometrial adenocarcinoma. In each case, upon the patient's written consent, the LBC sample was acquired after 3 months following the primary conventional PAP‐smear diagnosis. HPV test and methylation test were performed. Subsequently, histologic diagnosis information was collected for cytology–histology correlation purposes. Only cases with histologically confirmed diagnoses of HPV‐associated EA and AIS were included in further analysis.

### 2.1 HPV Testing

For HPV genotyping, DNA was isolated from 2 mL of LBC cervical samples using the QIAsymphony DNA Mini Kit (Qiagen, Hilden, Germany) according to the manufacturer's instructions. The HPV genotyping was performed using commercial assay Anyplex II HPV28 (Seegene), according to the manufacturer's instructions, and two sets of in‐house PCR, one PCR targeting the E1 gene of a broad spectrum of HPV types [13] and the second type‐specific PCR targeting E6/E7 oncogenes of six clinically important HPV types, namely, 16, 18, 31, 33, 35 and 45 [14].

### 2.2 Methylation Testing

Detection of methylation silencing of tumour‐suppressor genes was performed by multiplex real‐time methylation‐specific PCR using either the commercial QIAsure kit (Qiagen) (since 2018) or its first version—PreCursor–M Kit (Self–Screen) (from 2016 until 2017). Both were preceded by bisulfite conversion using the EZ DNA Methylation TM Kit (ZYMORESEARCH). The test targets the promoter area of two genes: *FAM19A4* (Family With Sequence Similarity 19 Member A4, C‐C Motif Chemokine Like), which is an alias for the *TAFA4* gene (Chemokine Like Family Member 4), located at 3p14.1. This protein is believed to function as a brain‐specific chemokine or neurokine that acts as a regulator of immune and nervous cells. The second gene, *miR124‐2*, is located at 8q12.3 and is involved in post‐transcriptional regulation of gene expression. A sample was considered hypermethylated if at least one detected marker was methylated above a specified threshold. According to the manufacturer's protocol, no further stratification of individual genes was to be provided.

## 3 Results

Altogether, 770 conventional PAP smears signed out as cytologically severe/significant glandular lesions (AGC–NEO+) were identified. In total, 286 LBC samples were collected from the study. HPV and methylation testing were successfully performed in 270 and 236 samples, respectively. Follow‐up biopsy information was available for 305 of 770 cases (40%). Complete data for inclusion in the study, that is, Pap test results, LBC sample molecular testing and follow‐up biopsy, were available for 168 patients. The diagnosis of HPV‐associated AIS or HPV‐associated invasive EA was histologically confirmed in 85 (51%) patients (AIS in 46 cases, EA in 39 cases). Samples from these patients were further analysed. HPV genotyping revealed 20 different HPV types and a multi‐infection rate of 29% (Table 1).

**TABLE 1:** A spectrum of 20 different HPV types identified in 85 cervical LBC samples harbouring HPV‐associated endocervical adenocarcinoma in situ (*n* = 46) and HPV‐associated endocervical adenocarcinoma (*n* = 39) with a multi‐infection rate of 29%.

HPV type	16	18	31	33	39	42	44	45	52	53	54	56	59	61	66	68	70	73	81	82
Number of cases	26	47	4	3	3	4	1	10	2	3	1	3	1	2	1	3	5	2	1	1

HPV types 16 and 18 were detected in 84% (72/85) of lesions, HPV type 45 in 12% (10/85)—five of which were single HPV type infections and five co‐infections (with HPV 16 and 45 being present in one case). Overall, HPV types 16, 18 and/or 45 were present in 96% (82/85) of cases (Supporting Information). The methylation test was positive in 95.3% (81/85) of cases, negative in 3.5% (3/85, AIS in two cases, EA in one case) and one sample (1.2%) was unanalysable (Supporting Information).

## 4 Discussion

The study uses the promoter methylation test targeting *FAM19A4 (TAFA4)* and *miR124‐2* genes, as this was the only methylation test available in the Czech Republic at the time of study initiation in 2016. Since then, the method has been widely used in Europe, and its performance for cervical squamous cell carcinoma was recently reviewed [12]. To the best of our knowledge, this is the first prospective study examining the sensitivity of methylation tests in identifying/detecting HPV‐associated AIS and EA [12]. Regarding the characteristics of the studied population, the dominance of HPV types 16/18 in studied AIS+ lesions in Czech women, representing (41/46) 89%, is in line with 86% reported in 2015 by the other group as national data on the HPV spectrum of 37 AIS+ lesions [15] and with 92% explicitly reported for the AIS only in the American population [10]. It differs from 74.1% reported for AIS+ cases with HPV types 16, 18, 45 and 52 detected in China Guangzhou area patients [16]. We acknowledge low complete study data retrieval, which may be the source of bias. Consequently, this study does not aim to establish methylation test specificity and other parameters relevant to test implementation in a population‐wide cervical cancer screening programme, and we do not comment on this topic. Instead, we focus solely on sensitivity based on the diagnostic gold standard of histologic tissue examination and show that the methylation test of the LBC sample is positive in 95.3% of histologically confirmed AIS+ cases. Its performance is close to the 99% accuracy of HPV tests reported for AIS previously [10].

Cytology alone accurately identifies approximately 50% of AIS+ cases, as previously noted by Miller [9] and Conrad [6], with 10/24 and 23/43 cases identified, respectively. Also, Niu [17] retrospectively analysed 72 AIS and 48 EA cases and reported sensitivity rates of 44.4% and 77.1% for detecting AIS and EA, respectively. Recently, it was reported based on samples from the Malmo area population in Sweden that the sensitivity of the FAM19A4/*miR124–2* methylation test is 51% (19 of 37 LBC samples) for histologically confirmed AIS and 70% (7 of 10 LBC samples) for EA [18]. The reliability of those findings and the power of conclusions are limited by a low number of EA cases and by the fact that the study was based on analysis of leftover LBC samples frozen at −80°C for more than 10 years (sampled and frozen in 2007–2012). The percentage of methylation‐positive LBC samples may vary when comparing freshly collected and stored LBC samples, as was already discussed [19]. Our current prospective study is focused on histologically confirmed AIS+ cervical lesions. Based on freshly collected LBC samples, it documents that in the ancillary testing of cervical cancer screening LBC samples, the methylation test shows higher sensitivity and reliably identifies 95.3% of the AIS+ lesions compared to 58.6% of HSIL lesions reported previously [19]. Simultaneously, the longitudinal study among 1040 HPV‐positive women enrolled in the POBASCAM screening trial in the Netherlands reported that, compared to a cytology negative (<ASCUS) result at enrolment, a negative *FAM19A4/MIR124‐2* methylation test indicated a lower risk of cervical cancer incidence over a 14‐year follow‐up period (Risk Ratio = 0.74, 95% CI:0.16–1.40) [20]. Further, the study by Zummeren showed that only (4/35) 11% cytologically negative cases (NILM) were methylation positive [21]. In our opinion, those findings allow the methylation test to be used in some unique clinical settings. Namely, if AGC–NEO+ Pap test results indicate the possibility of severe cervical glandular lesions in HPV‐positive pregnant women and young nulliparas. Chronic inflammation and IUD can sometimes produce endocervical mucosal changes, imitating AIS and EA cytologically [22, 23] and even histologically [24, 25, 26, 27, 28, 29, 30]. An ancillary methylation test could solve the challenge of cytological differential diagnosis. Its negativity would argue for the reactive nature of detected cytological changes and make the diagnosis of HPV‐associated AIS and EA less likely. Consequently, that could lead to different clinical management of the patient, avoiding mandatory confirmatory colposcopy‐oriented biopsy or small cone (appropriate only during the first trimester of pregnancy) [31] that precedes the fertility‐sparing surgical procedure at a specialised centre [32, 33, 34].

## 5 Conclusion

In our experience, within the context of ancillary testing of cervical cancer screening LBC samples, the methylation test demonstrates a high sensitivity of 95.3% for AIS+ lesions. This argues for further investigation into its performance characteristics and consideration of its use, especially as a pre‐surgical triaging test in sensitive cases if the AIS+ lesion is suspected in pregnant women or nullipara.

## Author Contributions

**Iva Kinkorová Luňáčková**, and **Ondrej Ondič** contributed equally by conceptualisation, project administration, data curation, writing draft and review. **Jana Němcová** molecular studies, methodology, manuscript review, data curation. **Kateřina Černá** molecular studies, methodology, data curation, manuscript review. **Jiří Bouda** funding acquisition, conceptualisation, methodology, data acquisition, curation and validation, manuscript review. **Jan Chytra** methodology, data acquisition, curation and validation, manuscript review.

## Ethics Statement

All clinical samples and data were obtained and used in accordance with protocols approved by the Institutional Ethics Boards of the contributing institution and with informed patient consent.

## Conflicts of Interest

The authors declare no conflicts of interest.

## Supporting information


**Table S1** HPV types, methylation status and histologic diagnoses in 85 Czech women diagnosed with HPV‐associated endocervical adenocarcinoma in situ (AIS) (*n* = 46) and invasive endocervical adenocarcinoma, HPV‐associated (ECA) (*n* = 39); p– punch biopsy, c—curettage, HYE—hysterectomy, LVI—lymphovascular invasion, NA—non‐analysable

## Data Availability

Data sharing is not applicable to this article as no new data were created or analyzed in this study.
