# Supplementary material for: The Methylation Test Is Highly Sensitive for HPV‐Associated Endocervical Adenocarcinoma and Could Be Helpful as a Cytological Ancillary Test in Women With a PAP‐Smear Diagnosis of Severe Glandular Lesion (AGC–NEO+)
Source: Cytopathology. 2025 Jun 13;36(6):568–71. doi: 10.1111/cyt.70001 (PMC12501819; doi:10.1111/cyt.70001)
Supplement: Supplementary file 1 — Table S1 HPV types, methylation status and histologic diagnoses in 85 Czech women diagnosed with HPV‐associated endocervical adenocarcinoma in situ (AIS) (n = 46) and invasive endocervical adenocarcinoma, HPV‐associated (ECA) (n = 39); p– punch biopsy, c—curettage, HYE—hysterectomy, LVI—lymphovascular invasion, NA—non‐analysable [file CYT-36-568-s001.docx]

**Supplementary material**

**Title:** The methylation test is highly sensitive for HPV-associated endocervical adenocarcinoma and could be helpful as a cytological ancillary test in women with a PAP smear diagnosis of severe glandular lesion (AGC-NEO+).

**Authors:** Iva Kinkorová Luňáčková^1^, Ondrej Ondič^1,2^, Jana Němcová^1,2^, Kateřina Černá^1,2^, Jan Chytra^3^, Jiří Bouda^3^

**Table S1** HPV types, methylation status and histologic diagnoses in 85 Czech women diagnosed with HPV– associated endocervical adenocarcinoma in situ (AIS) (n=46) and invasive endocervical adenocarcinoma, HPV associated (ECA) (n=39); p– punch biopsy, c– curretage, HYE– hysterectomy, LVI – lymphovascular invasion, NA– non–analysable

| **ID** | **HPV type** | **Methylation status** | **Histology** |
| --- | --- | --- | --- |
| 1 | 16 | Positive FAM+miR | AIS |
| 2 | 16 | Positive FAM+miR | AIS |
| 3 | 16 | Positive FAM+miR | AIS |
| 4 | 16 | Positive FAM+miR | AIS |
| 5 | 16 | Positive FAM+miR | AIS |
| 6 | 16 | Positive FAM+miR | AIS and co-occurring HSIL (CIN 2-3) |
| 7 | 16 | Positive FAM+miR | ECA, FIGO stage IB1 (pattern B), LVI negative |
| 8 | 16 | Positive FAM+miR | ECA, endometroid type, FIGO IB2 pN0 (0/15) cM0, G2, HPV16+ |
| 9 | 16 | Positive FAM+miR | ECA, endometrioid type, FIGO IB2 |
| 10 | 16 | Positive FAM+miR | ECA |
| 11 | 16 | Positive FAM+miR | ECA ( pattern A), pT1a1, LVI negative |
| 12 | 16 | Positive FAM+miR | ECA, usual, Pattern A (c), HYE negative. |
| 13 | 16,68 | Positive FAM+miR | ECA, post LEEP, pT1b |
| 14 | 18 | Positive miR | AIS |
| 15 | 18 | Positive miR | AIS, minimal |
| 16 | 18 | Positive FAM+miR | AIS |
| 17 | 18 | Positive FAM+miR | AIS (p) and in LEEP /pregnant patient/ |
| 18 | 18 | Positive FAM+miR | AIS (c); HYE-negavite |
| 19 | 18 | Positive FAM+miR | AIS (c) and co-occurring HSIL, HYE negative. |
| 20 | 18 | Positive FAM+miR | AIS and co-occurring HSIL (CIN3) |
| 21 | 18 | Positive FAM+miR | AIS and co-occurring HSIL (cone biopsy), reconisation - negative |
| 22 | 18 | Positive FAM+miR | AIS |
| 23 | 18 | Positive FAM+miR | AIS and co-occurring HSIL, reconisation - negative |
| 24 | 18 | Positive FAM+miR | AIS and co-occurring HSIL |
| 25 | 18 | Positive FAM+miR | AIS and co-occurring HSIL (CIN3) |
| 26 | 18 | Positive FAM+miR | AIS and co-occurring HSIL |
| 27 | 18 | Positive FAM | AIS |
| 28 | 18 | Positive FAM | AIS |
| 29 | 18 | Positive FAM | AIS |
| 30 | 18 | Negative | AIS and co-occurring HSIL (CIN3) |
| 31 | 18 | Positive FAM+miR | ECA, FIGO IB1 |
| 32 | 18 | Positive FAM+miR | 164 651/18 adenosquamous carcinoma, pT2b pN1(sn) M0, grade 2, FIGO IIIB |
| 33 | 18 | Positive FAM+miR | ECA, pT1b1, FIGO IB1, p16+, HPV 18, HYE |
| 34 | 18 | Positive FAM+miR | ECA (c), FIGO at least IA2; HYE – AIS remnants |
| 35 | 18 | Positive | ECA and AIS, pattern B, Lymph node - negative, trachelectomy |
| 36 | 18 | Positive | p-HSIL+AIS+SMILE; c – ECA, pT1a1; HYE negative |
| 37 | 31 | Positive FAM+miR | AIS |
| 38 | 45 | Positive FAM+miR | AIS |
| 39 | 45 | Positive FAM+miR | ECA, pattern A, co-occurring HSIL (CIN3), HPV 45 |
| 40 | 16,39,70 | Positive FAM+miR | AIS and co-occurring HSIL (CIN2, CIN3) |
| 41 | 16,56,70 | Positive FAM+miR | ECA, pT1b1 pN0 (0/21) cM0, co-occurring HSIL |
| 42 | 16,66,44,54 | Positive FAM+miR | ECA, pT1b |
| 43 | 18,31,70 | Positive FAM+miR | AIS and co-occurring HSIL- c; HYE- residual AIS and HSIL |
| 44 | 18,42 | Positive FAM+miR | AIS |
| 45 | 18,52,53 | NA | AIS and HISL (CIN 3) in cone biopsy |
| 46 | 18,59 | Positive FAM+miR | AIS |
| 47 | 18,68 | Positive FAM+miR | AIS |
| 48 | 45 (73) | Positive FAM+miR | ECA, pattern B, no LVI |
| 49 | 45,53,42 | Positive FAM | AIS |
| 50 | 56 (weak) | Positive FAM + miR | ECA, biopsy no: 1103871/20 |
| 51 | HPV 18 | Negative (double isolation) | ECA, usual, FIGO IA1 and AIS |
| 52 | HPV 18,39 | Positive | ECA |
| 53 | HPV 18,42,81 | Positive FAM+miR | AIS in cone biopsy, HYE negative |
| 54 | HPV+ (one of 16/18/45) | Positive FAM, miR - NA | AIS |
| 55 | HPV16 | Positive FAM+miR | ECA, with LVI, pT1b1N0M0 |
| 56 | HPV16 | Positive FAM+miR | ECA, pattern A and minor B, 17x27x12 mm, no LVI |
| 57 | HPV16 | Positive FAM+miR | ECA, pattern A, FIGO IB1 |
| 58 | HPV16 | Positive FAM+miR | ECA endometroid type, pN0 (20) |
| 59 | HPV16 | Positive FAM+miR | ECA, pTIB1, pN0 (20) |
| 60 | HPV16 | Positive FAM + miR | ECA |
| 61 | HPV16,18 | Positive FAM+miR | AIS in cone biopsy, HYE negative |
| 62 | HPV16,45,61 | Negative | AIS |
| 63 | HPV16,68 | Positive FAM+miR | AIS, p16 positive |
| 64 | HPV16,70 | Positive FAM+miR | AIS and co-occurring LSIL |
| 65 | HPV18 | Positive miR | ECA and AIS |
| 66 | HPV18 | Positive FAM+miR | ECA pattern A; ( size 6x2x1 mm) and AIS |
| 67 | HPV18 | Positive FAM+miR | ECA |
| 68 | HPV18 | Positive FAM+miR | ECA; microinvasive usual, pattern A and AIS |
| 69 | HPV18 | Positive FAM + miR | ECA, Exophytic mucin-producing |
| 70 | HPV18 | Positive FAM + miR | ECA, mucinous, intestinal type, grade 2-3, (pattern C) |
| 71 | HPV18 | Positive FAM + miR | ECA, villoglandular adenocarcinoma, grade 1 |
| 72 | HPV18 | Positive FAM | ECA, FIGO IB1, no LVI |
| 73 | HPV18 | Positive FAM + miR | AIS in cone biopsy, HYE negative |
| 74 | HPV18,31 | Positive FAM | AIS and HSIL with glandular extension - p |
| 75 | HPV18,52,70 | Positive FAM+miR | AIS |
| 76 | HPV18,53,42 | Positive FAM+miR | AIS + microinvasive squamous ca + HSIL (CIN 3) |
| 77 | HPV18,61 | Positive FAM+miR | ECA, pattern C, FIGO IB1 and AIS |
| 78 | HPV18,73 | Positive FAM + miR | AIS |
| 79 | HPV18,31(weak) | Positive FAM + miR | ECA, intestinal type |
| 80 | HPV33,39,56,82 | Positive FAM+miR | ECA, intestinal type (gastric-like) |
| 81 | HPV33,45 | Positive FAM+miR | AIS |
| 82 | HPV45 | Positive FAM+miR | ECA, pattern B, FIGO IB1 |
| 83 | HPV45 | Positive FAM+miR | ECA, FIGO lB1(6x7x7mm) pN0 (3sln/0) M0, LVI- negative |
| 84 | HPV45 | Positive | ECA and AIS and HSIL, FIGO IB1 N0 (SLN) M0; HYE negative |
| 85 | HPV45,33 | Positive FAM + miR | AIS-c, HYE negative |
